# An evaluation of the protective role of vitamin C in reactive oxygen species-induced hepatotoxicity due to hexavalent chromium in vitro and in vivo

**DOI:** 10.1186/s12995-017-0161-x

**Published:** 2017-06-15

**Authors:** Xiali Zhong, Ming Zeng, Huanfeng Bian, Caigao Zhong, Fang Xiao

**Affiliations:** 1 Department of Health Toxicology, Xiangya School of Public Health, Central South University, NO. 238 Shangmayuanling Road, Kaifu District, Changsha, 410078 Hunan People’s Republic of China; 2 Shajing Institution of Health Supervision of Baoan District, Shenzhen, 518104 People’s Republic of China

**Keywords:** Hexavalent chromium [Cr (VI)], Reactive oxygen species (ROS), Hepatotoxicity, Vitamin C (Vit C), Mitochondrial respiratory chain complex I (MRCC I)

## Abstract

**Backgroud:**

Drinking water contamination with hexavalent chromium [Cr (VI)] has become one of the most serious public health problems, thus the investigation of Cr (VI)-induced hepatotoxicity has attracted much attention in recent years.

**Methods:**

In the present study, by determining the indices of hepatotoxicity induced by Cr (VI), the source of accumulated reactive oxygen species (ROS), and the protective effect of the antioxidant Vitamin C (Vit C), we explored the mechanisms involved in Cr (VI)-induced hepatotoxicity in vitro and in vivo.

**Results:**

We found Cr (VI) caused hepatotoxicity characterized by the alterations of several enzymatic and cytokine markers including aspartate aminotransferase (AST), alanine aminotransferase (ALT), interleukine-1β (IL-1β), and tumor necrosis factor-α (TNF-α), etc. ROS production after Cr (VI) exposure was origins from the inhibition of electron transfer chain (ETC) and antioxidant system. Vit C inhibited ROS accumulation thus protected against Cr (VI)-induced hepatotoxicity in L-02 hepatocytes and in the rat model.

**Conclusions:**

We concluded that ROS played a role in Cr (VI)-induced hepatotoxicity and Vit C exhibited protective effect. Our current data provides important clues for studying the mechanisms involved in Cr (VI)-induced liver injury, and may be of great help to develop therapeutic strategies for prevention and treatment of liver diseases involving ROS accumulation for occupational exposure population.

## Background

Chromium (Cr) is commercially used in various industrial processes such as leather tanning, stainless steel welding and chrome plating [1]. The pollution of hexavalent chromium [Cr (VI)] has become one of the most serious public health problems worldwide and the serious pollution incidents have dramatically increased in the last few years. The adverse health effects following occupational or accidental exposures of Cr (VI) and its compounds include a range of slight gastrointestinal reactions such as nausea and vomiting to more serious effects including hepatic damage, primary liver cancers, and even death [2]. Now we have to pay attention that not only the Cr-operating workers, but also the general population may also be at risk because Cr (VI) and the compounds are now widespread in our intimately related environment even in the food [3]. It is known that Cr (VI) enters the target cells and then undergoes metabolic reduction to pentavalent chromium [Cr (V], tetravalent chromium [Cr (IV)] and trivalent chromium [Cr (III)], causing the accumulation of reactive oxygen species (ROS) [4]. ROS, including hydroxyl radicals (OH·), hydrogen peroxide (H_2_O_2_), and superoxide anion radical (O_2_·^−^), are the molecules that contain an odd number of electrons. The main sources of intracellular ROS are enzymatic reactions, nicotinamide adenine dinucleotide phosphate-oxidase (NADPH oxidase), and mitochondrial respiration [5]. Mitochondrial respiratory chain complexes (MRCCs), whose inhibition may cause the escape of electrons from electron transfer chain (ETC), are the most important source of heavy metal-induced ROS accumulation [6]. The major sites of ETC for ROS production remained controversial, and it is reported by others that MRCC III from the ETC is the major site for ROS production [7]. The members of antioxidant system including superoxide dismutase (SOD) and glutathione (GSH) are known as ROS scavenging enzymes whose function are associated with the elimination of excess ROS. ROS play an important role in various cellular signaling processes at low levels while exert a cytotoxic effect by damaging macromolecules such as proteins, lipids and nucleic acids. The reduction of Cr (VI) results in the formation of free radicals which induce a cascade of cellular events including apoptosis, genotoxicity and carcinogenicity, but the related mechanisms of the accumulated free radicals still remain unclear. In the present study we hypothesize that the accumulation of free radicals after Cr (VI) exposure is associated with both the burst generation and the decreased elimination of ROS.

Since there is accumulating evidence indicating a possible causative involvement of ROS in liver injury [8], the anti-oxidative therapy may be of great importance for the established Cr (VI)-associated liver diseases. Vitamin C (Vit C), also known as ascorbic acid, is necessary for the body and widely found in fruits and vegetables. Vit C plays a role as an essential coenzyme in the oxidative stress (OS) pathways, and is an important antioxidant and ROS scavenger. Therefore, Vit C is potentially useful as a therapeutic agent in the treatment of the disorders that associated with free radicals. The liver, an important body organ for its involvement in the biotransformation of various xenobiotics, plays crucial roles in metal homeostasis and detoxification. Although Cr (VI) has been reported to induce hepatotoxicity and natural and synthetic antioxidants have been shown to exert protective effects, the related molecular and cellular mechanisms as well as the potential anti-hepatotoxic protective effect of Vit C on Cr (VI)-induced hepatotoxicity both in vitro and in vivo remain to be fully elucidated. Therefore, we aimed to explore the underlying mechanism of Cr (VI)-induced hepatotoxicity and the possibility that the administration of Vit C would have a beneficial effect on Cr-induced hepatic injuries for the occupation exposure population. The present work was undertaken to study the liver injury by detecting the enzymatic and cytokine markers, to examine the possible sources of the elevated ROS, and to investigate the protective effect of Vit C on Cr (VI)-induced hepatotoxicity both in L-02 hepatocyte and in the rat model.

## Materials and methods

### Cell line

Human L-02 hepatocyte line was obtained from Type Culture Collection of Chinese Academy of Sciences, Shanghai, China. Cells were cultured as previously described [9].

### Animals

The adult Sprague-Dawley (SD) rats aged about 2 months with the average body weight of 180 ± 20 g were purchased from the animal center of Central South University (Changsha, Hunan, China). All rats were housed at the temperature of 22 ± 2 °C and a humidity of 55 ± 5% in a 12h light/dark cycle in standard clear plastic cages with food and water. All animal experiments were performed in accordance with the guidelines of China Council on Animal Care and Use. All animal procedures carried out in this study were reviewed, approved, and supervised by the Animal Care and Use Committee of Central South University.

### Animal experiment design

Sixty SD rats were randomly divided into 6 groups, each with 10 animals. Group 1 was the control group, and received normal saline (NS); group 2 was treated with low dose of Cr (VI) (8.84 mg/kg.Bw) (potassium dichromate (K_2_Cr_2_O_7_) was dissolved in NS and then was configured to various test doses); group 3 received high dose of Cr (VI) (17.68 mg/kg.Bw); group 4 received Vit C alone (500 mg/kg.Bw); group 5 was treated with the combination of Vit C (500 mg/kg.Bw) plus Cr (VI) (8.84 mg/kg.Bw); group 6 was treated with the combination of Vit C (500 mg/kg.Bw) plus Cr (VI) (17.68 mg/kg.Bw). All rats were given the drugs by gavage at a dose of 0.5 ml/100 g body weight daily for a week (7 consecutive days). Group 1, 2 and 3 were firstly treated with NS by gavage, half an hour later, group 1 received NS and group 2 and 3 received Cr (VI); group 4, 5 and 6 were firstly treated with Vit C by gavage, half an hour later, group 4 received NS and group 5 and 6 received Cr (VI).

### Sample collection and preparation

One week later and at the end of the experiment, the urine and stool of the last 24 h treatment of all rats were collected. The urine was precipitated to remove residue, and the stool was dried to constant weight. Blood samples (4 ml of each rat) collected from the femoral artery were allowed to coagulate for 30 min and centrifuged at 2500 rpm for 15 min to separate the serum for biochemical analysis as described below. The rats were sacrificed using ether anesthesia. For each rat, liver specimen (1 g) was collected and then was suspended in ice-cold NS and homogenized in a polytron homogenizer to obtain 10 ml liver tissue suspension. All the samples were stored at −80 °C for further analysis.

### Materials

K_2_Cr_2_O_7_ and Vit C were purchased from Sigma (St. Louis, MO, USA). RPMI-1640 culture medium, fetal bovine serum (FBS), and trypsin were obtained from Solarbio (Beijing, China). All chemicals and solvents were of analytical grade or the best pharmaceutical grade.

### The detection of enzymatic makers of liver injury

The supernatant from treated hepatocytes and serum samples from rats in groups 1 to 6 were analyzed for aspartate aminotransferase (AST) and alanine aminotransferase (ALT) activities spectrophotometrically using the detection kits (Jiancheng Institute of Biological Products, Nanjing, China). The experiments were performed according to the manufacturer’s protocols.

### The detection of cytokines and LTB4 levels

The levels of leukotriene B4 (LTB4) and the cytokines including interleukine-1β (IL-1β), tumor necrosis factor-α (TNF-α), interferon-γ (IFN-γ), and interleukine-10 (IL-10) were examined using the enzyme linked immunosorbent assay (ELISA) detection kits (Huamei Institute of Biological Products, Wuhan, China). The experiments were performed according to the manufacturer’s protocols.

### ROS detection

ROS levels were evaluated using fluorescent probe 5-(and 6)-chloromethyl-2′, 7′-dichlorodihydrofluorescein diacetate (CM-H2DCFDA, Molecular Probers, USA). Briefly, after the treatment of various compounds as indicated in the legends to figures, cells of each group were incubated with 10 μM CM-H2DCFDA and analyzed by fluorescence microscope and flow cytometry (Ex 485 nm and Em 535 nm). 2′, 7′-dichlorofluorescein (DCF) is the oxidized product of CM-H2DCFDA. Intracellular ROS level was considered to be directly proportional to the fluorescence intensity of the oxidized product DCF after CM-H2DCFDA treatment. Three independent experiments were performed for each assay condition.

### Superoxide anion production detection

Superoxide anion production was assessed by dihydroethidium (DHE) staining. The cells were cultured and treated as above. After rinsed twice with phosphate buffered saline (PBS), the cells were incubated in the dark with 5 μM DHE (Sigma-Aldrich, St Louis, MO) for 30 min. In the presence of superoxide anion, DHE can be oxidized to ethidium bromide (EtBr) (Ex 488 nm and Em 610 nm) which expressing red fluorescence. Thus the amount of EtBr is well correlated to the level of cellular superoxide anion. Superoxide anion in the cells was analyzed by flow cytometry and presented by the percentage of positively staining cells.

### Mitochondria isolation

Mitochondria were isolated as described previously [10]. Cells were washed twice with cold PBS, and resuspended with 5 ml buffer A (250 mM sucrose, 20 mM 4-(2-hydroxyethyl)-1-piperazineethanesulfonic acid (HEPES), 10 mM KCl, 1.5 mM MgCl_2_, 1 mM ethylene diaminetetra acetic acid (EDTA), 1 mM ethylene glycol-bis (2-aminoethylether)- N, N, N ′, N ′-tetraacetic acid (EGTA), 1 mM dithiothreitol, 0.1 mM phenylmethylsulfonyl fluoride, pH 7.5). Cells were homogenized and centrifuged twice at 750×*g* for 10 min. Mitochondria pellets were obtained after centrifugation at 10,000×*g* for 15 min. Isolated mitochondria were used immediately for the measurement of complexes activity. In order to confirm the purity and functionality of the purified mitochondria, transmission electron microscope was used to observe the ultra-structure at magnification of 300,000 times and Clark-type oxygen electrode was used to detect the respiratory function.

### Measurement of MRCC activities

The activities of MRCC I-IV were determined using MRCC activity assay kits (Genmed Scientifics, shanghai, China) and were quantified using an UV-9100 spectrophotometer. MRCC I (Nicotinamide adenine dinucleotide (NADH) CoQ oxidoreductase) activity was measured following the oxidation of NADH at 340 nm and expressed as nmol oxidized NADH/min/mg prot; MRCC II (succinate: 2, 6-Dichloroindophenol (DCIP) oxireductase) activity was measured following the reduction of DCIP at 600 nm and expressed as nmol reduced DCIP/min/mg prot; MRCC III (ubiquinol: cytochrome c (Cyt c) reductase) activity was measured following the reduction of Cyt c at 550 nm and expressed as nmol reduced Cyt c /min/mg prot, and MRCC IV (Cyt c oxidase) activity was measured following the oxidation of Cyt c at 550 nm and expressed as nmol oxidized Cyt c/min/mg prot. All measurements were performed at least three times.

### Real-time quantitative PCR

Total RNA was extracted from L-02 hepatocytes treated with different concentrations of Cr (VI) using the RNeasy Mini Kit (QIAGEN, Hilden, Germany). Then the total RNA (5 μg) from each treatment group was reverse-transcribed by the PrimeScript RT reagents kit (Takara, Dalian, China) according to the standard protocol. cDNAs were analyzed immediately for Real-time PCR assay using SYBR®Premix Taq™ (Takara, Dalian, China) with Applied Biosystems 7900HT Fast Real-Time PCR System (Applied Biosystems, Inc., Foster City, CA, USA) to observe the mRNA levels of targeted genes. The primer sequence of MRCC I [NADH dehydrogenase [ubiquinone] iron-sulfur protein 3 (NDUFS3)]: 5′- atgttgcccaaactggtctc −3 (forward primer), 5′- tcactgccttcccagagagt −3′ (reverse primer).

### Measurement of GSH, SOD, Trx, MDA cellular levels and protein levels

The assessments of the GSH, SOD, and malondialdehyde (MDA) levels were conducted by using the standard kits (Jiancheng Bioengineering Institute, Nanjing, China). And the examination of thioredoxin (Trx) level was also using the kit (Huamei Bioengineering Institute, Wuhan, China). GSH level was examined by the amount of total non-protein sulfhydryl groups, SOD level was examined based on the inhibitory effect of SOD on nitro blue tetrazolium (NBT), Trx level was examined by a double antibody sandwich ELISA method, and MDA level was examined by thiobarbituric acid reactive substances (TBARS).

Protein levels were determined by Western Blotting. Cell lysate was prepared by lysing the cells and then the protein was electrophoretically transferred onto polyvinylidene difluoride (PVDF) membranes and immunoblotted with the antibodies GSH-1 (H-41) (sc-292,189), SOD-1 Antibody (FL-154) (sc-11,407), and Trx (FL-105) (sc-20,146) (Santa Cruz Biotechnology, USA). After incubated with second antibodies, the membranes were developed with the detection system and exposed to films.

### Measurement for cell survival rate

Three-(4,5-dimethylthiazol-2yl-)-2,5-diphenyl tetrazolium bromide (MTT) assay was used to evaluate cell survival rate. Briefly, the hepatocytes were seeded at a density of 1 × 10^4^ cells/well in the 96-well plate. Vit C of indicated final concentrations were added to the cultures. Control cells and medium controls without cells received DMSO. The cells were incubated at 37 °C in 5% CO_2_ saturated atmosphere and then were washed twice with PBS. Cells were treated with 5 μl 5 mg/ml MTT solution for additional 4 h at 37 °C, and then were lysed in PBS containing 20% Sodium dodecyl sulfate (SDS) and 50% N, N-dimethylformamide (pH 4.5). MTT conversion was quantified by a multiwell ELISA reader Versamax (Molecular Devices, Sunnyvale, CA, USA) at 570 nm.

### Histopathological examination of liver

Liver tissue samples were fixed overnight with 10% neutral buffered formalin and then were rinsed for 2 h using running water. The tissues were dehydrated with different concentrations of ethanol and dimethylbenzene and then were embedded in paraffin, sectioned at a thickness of 4 μm, and stained with hematoxylin and eosin (H&E). The specimens were then examined under light microscopy.

### Measurement of Cr content

The chromium contents of the samples were determined using the flame atomic absorption spectrometry (F-AAS) method as described earlier [11]. Briefly, prepare chromium standard solution (10 μg/l) and add with 1% spectra pure HNO_3_. Then determine the absorbance of different concentrations of standard chromium solution (0, 0.2, 0.5, 1.0, 2.0, 4.0 μg/l) and draw the standard curve. Cr contents of stool, urine, liver and plasma samples were then measured at a wavelength of 357.9 nm.

### Measurement of free radical scavenging capacity

The assay was based on benzoic acid hydroxylation method with slight modification. Briefly, in a colorimetric tube, 20 μl FeSO_4_ (20 mM) and 20 μl EDTA (3 mM) were added. Then, 100 μl sample solution and 1840 μl PBS (pH 7.4) and 20 μl benzoic acid (10 g/l) were added to give a total volume of 2 ml. The reaction mixture was incubated in 37 °C water bath for 90 min. Then the fluorescence was measured at excitation/emission wavelengths of about 305/410 nm. Free radical scavenging capacity % = 100% × [Fs-(Ft-Fc)]/(Fs-Fc). Fs is the fluorescence intensity of the colorimetric tube without the adding of liver sample solution, Ft is the fluorescence intensity of the colorimetric tube with the adding of liver samples from different treatment groups, and Fc is the fluorescence intensity of the colorimetric tube without the adding of FeSO_4_ or liver sample solution.

### Statistical analysis

The results are expressed as mean ± standard deviation (SD). Normal distribution test and Levene’s test were performed for Equality of Variances. Significant differences were calculated by one-way analysis of variance (ANOVA) (data fitting normal distribution) or Kruskal-Wallis rank test (data not fitting normal distribution). All statistical analyses were performed using SPSS 19.0. The level of significance was set at *p* < 0.05.

## Results

### Cr (VI) induced hepatotoxicity in vitro

**Fig. 1:**
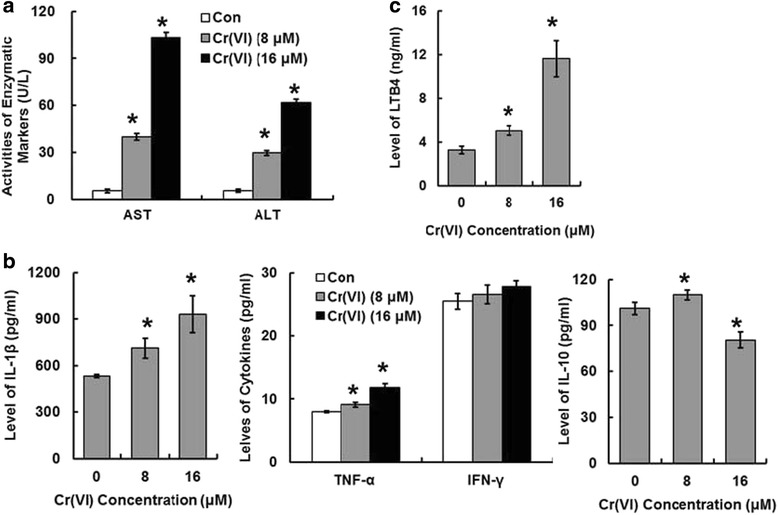
Cr (VI) induced hepatotoxicity in vitro. L-02 hepatocytes were treated with Cr (VI) (8 and 16 μM) for 24 h, then the cells were collected and the indexes of hepatotoxicity were determined. **a** The changes of activities of enzymatic markers AST and ALT. **b** The production of pro-inflammatory cytokines (IL-1β, TNF-α, IFN-γ and IL-10) after Cr (VI) exposure. **c** Effect of Cr (VI) exposure on LTB4 level. Data represent mean ± SD. *p < 0.05, compared with the control (untreated) group

AST and ALT levels are widely used enzymatic markers of hepatotoxicity. The Cr (VI) treatment groups (8 and 16 μM) showed considerable increase in AST and ALT activities compared with that of the control group (Fig.1a). IL-1β is a potent pro-inflammatory cytokine and IL-10 is an anti-inflammatory cytokine. TNF-α and IFN-γ are also the members of cytokine family that involved in systemic inflammation. IL-10 possesses a hepatic protective effect on proliferation, and it has been shown to inhibit the production of pro-inflammatory cytokines, such as TNF-α and IL-1β during the acute inflammation. As shown in Fig. 1b, after Cr (VI) exposure, IL-1β and TNF-α production were increased obviously compared with control, while IFN-γ level showed no obvious change. IL-10 level was increased at the Cr (VI) treatment concentration of 8 μM and decreased at 16 μM. Fig. 1c revealed that Cr (VI) induced increased level of LTB4.

### Cr (VI) induced ROS accumulation in the hepatocytes

**Fig. 2:**
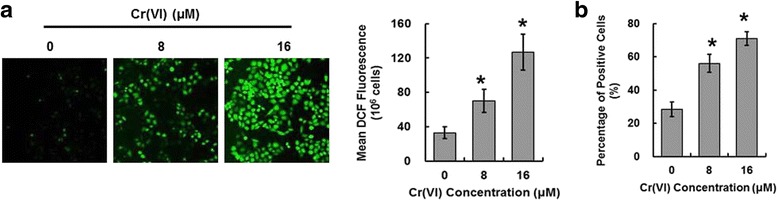
Cr (VI) induced ROS accumulation in the hepatocytes. The cells were treated as described in Fig. 1. **a** The DCF fluorescence intensity, corresponding to the level of ROS production, was detected. **b** Intracellular superoxide anion. The data shows the percentage and the fluorescence intensity of positive DHE staining cells from each group. Data represent mean ± SD. *p < 0.05, compared with the control group

ROS have been implicated in liver injury. To evaluate the effect of Cr (VI) treatment on intracellular ROS level, we performed the assay by utilizing fluorescent probe CM-H2DCFDA in L-02 hepatocytes treated with different concentrations of Cr (VI) (8 and 16 μM). We identified that compared with control group, Cr (VI) treatment induced higher level of fluorescence signals when observed under microscope (Fig. 2a, left panel). The values of the related DCF fluorescence of each group quantitated by flow cytometry were also shown (Fig. 2a, right panel). DCFH can be oxidized directly by Cr intermediates such as Cr (IV) and Cr (V), thus in the present study we also used DHE which is insensitive to Cr intermediates to monitor superoxide anion production. Superoxide anion is one of a group of molecules referred to as ROS. It has been confirmed that superoxide anion can be produced by both MRCCs and NADPH oxidase in a highly regulated manner and low amounts of superoxide anion plays critical roles in cell proliferation and apoptosis [12]. Superoxide anion production in the hepatocytes was assessed by DHE staining. We found that the percentage of positive staining cells was obviously higher in Cr (VI)-treated groups than that of the control group (Fig. 2b).

### The inhibition of MRCC I and antioxidant system were associated with Cr (VI)-induced ROS accumulation

**Fig. 3:**
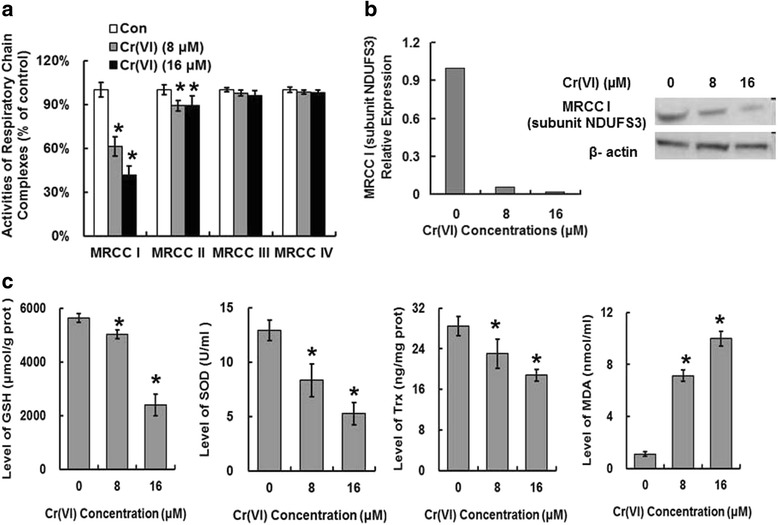
The inhibition of ETC and antioxidant system were associated with Cr (VI)-induced ROS accumulation. The cells were treated as described in Fig. 1. **a** The activities of MRCC I-IV. **b** The mRNA and protein levels of MRCC I subunit NDUFS3. **c** GSH, SOD, Trx, and MDA levels. Data represent mean ± SD. *p < 0.05, compared with the control group

The enzyme complexes of the ETC locate in the mitochondrial inner membrane and play central roles in energy metabolism and other physiological activities. We inferred that the MRCCs in ETC may be responsible for ROS overproduction because the inhibition of MRCCs could induce increased electron leakage from ETC. We examined the alterations of MRCC I-IV activities in purified mitochondrial fraction. MRCC I appeared to be the most affected one after Cr (VI) exposure. Although complex II was also altered to some extent, the change was not as significant as that of complex I. The activities of MRCC III and IV were not altered compared with control (Fig. 3a). The result suggested that MRCC I and II, especially the former may be the main target of Cr (VI) to induce mitochondrial ETC dysfunction and ROS accumulation. NDUFS3 encodes one of the iron-sulfur protein components of MRCC I. Mutations or inhibition of this gene are associated with MRCC I deficiency. The results shown in Fig. 3b revealed that Cr (VI) inhibited NDUFS3 at both mRNA and protein levels. GSH, SOD, and Trx are main antioxidative proteins that involved in ROS clearance. It is reported that some chemotherapeutic agents cause ROS-dependent cytotoxicity by down-regulating the expression of the antioxidative proteins to facilitate ROS over-production, thus we tested these proteins expressions in Cr (VI)-treated hepatocytes to confirm the resource of ROS. As shown in Fig. 3c, Cr (VI) decreased GSH, SOD and Trx levels in the dose-dependent manner. Cr (VI) also increased MDA level which suggested the occurrence of lipid peroxidation.

### Vit C inhibited ROS accumulation

**Fig. 4:**
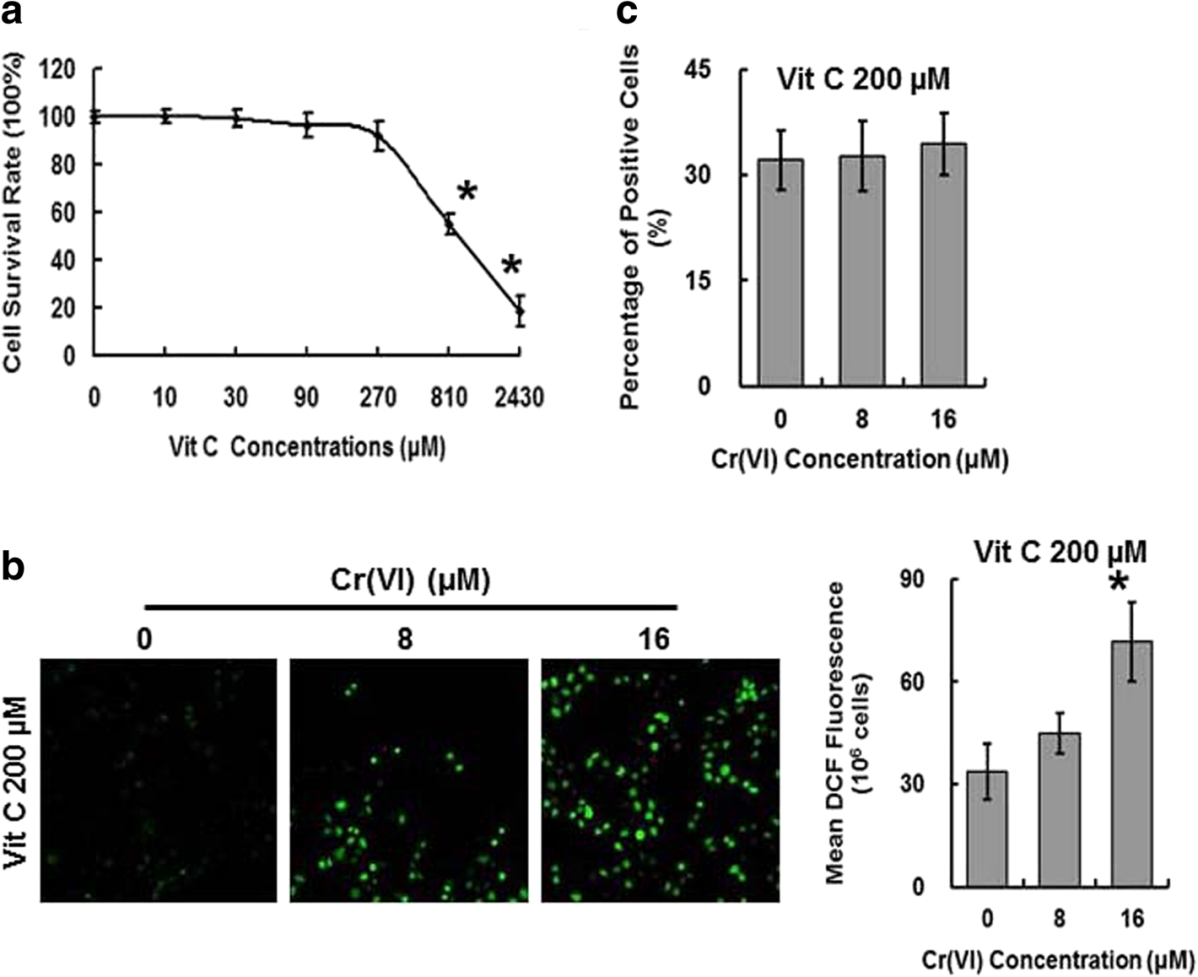
Vit C inhibited ROS accumulation. **a** The hepatocytes were treated with different concentrations of Vit C (0–2430 μM) for 2 h and then analyzed for cell survival rate. (B-C) The L-02 hepatocytes were pretreated with Vit C (200 μM) for 2 h and then were exposed to Cr (VI) (8 and 16 μM) for 24 h. ROS production assay (**b**) and intracellular superoxide anion production assay (**c**) were conducted. Data represent mean ± SD. *p < 0.05, compared with the control group

The potential role of free radicals in hepatotoxicity associated with Cr (VI) exposure suggests that antioxidant supplementation may mitigate Cr (VI)-induced toxicity, thus we utilized Vit C in the present study to decrease intracellular ROS level. We analyzed the effect of Vit C (0–2430 μM) on cell survival rate and chose the concentration of 200 μM for the following studies according to the MTT result showed in Fig. 4a. To confirm the antagonistic effect of Vit C on Cr (VI)-induced free radical accumulation, L-02 hepatocytes were pretreated with Vit C (200 μM) for 2 h and then were exposed to Cr (VI) (8 and 16 μM) for 24 h. ROS assay was then performed as described before. As shown in Fig. 4b, Vit C decreased the fluorescence signal levels under microscope and the DCF fluorescence values by flow cytometry, suggesting the inhibition of Cr (VI)-induced ROS production. The DHE staining also showed that Vit C inhibited superoxide anion production by decreasing the percentage of positive staining cells (Fig. 4c).

### Vit C protected against Cr (VI)-induced hepatotoxicity in vitro

**Fig. 5:**
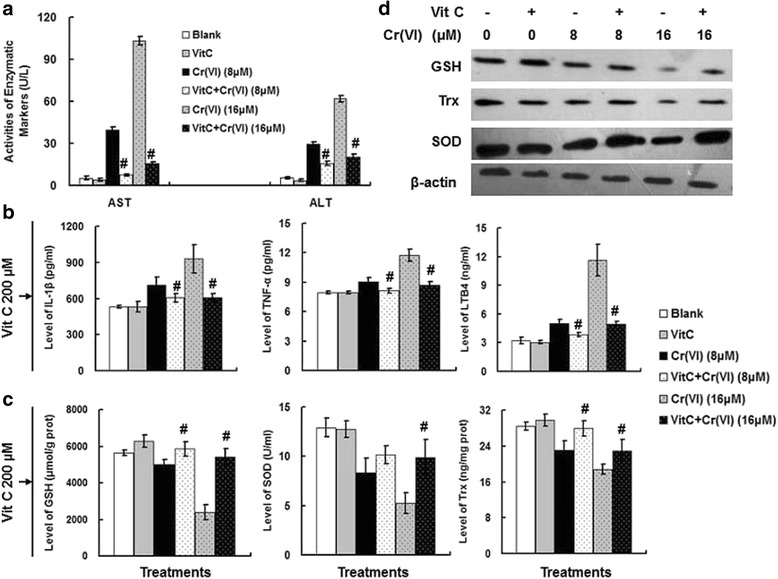
Vit C protected against Cr (VI)-induced hepatotoxicity in vitro. The cells were exposed to Cr (VI) (0, 8 and 16 μM) with or without the combination of 200 μM Vit C. **a** The changes of activities of enzymatic markers AST and ALT. **b** The levels of IL-1β, TNF-α and LTB4. **c** The levels of GSH, SOD, and Trx. **d** The protein expression levels of GSH, SOD, and Trx. Data represent mean ± SD. ^#^ *p* < 0.05, compared with Cr (VI) alone treatment (8 or 16 μM) group

The cells were exposed to Cr (VI) (0, 8 and 16 μM) with or without the pretreatment of 200 μM Vit C. Vit C significantly inhibited the increase of Cr (VI)-induced AST/ALT levels (Fig. 5a). We also examined effect of Vit C on other liver injury markers including IL-1β, TNF-α and LTB4 and obtained the similar results (Fig. 5b). Vit C also restrained Cr (VI)-induced antioxidant system dysfunction by inhibiting the decrease of GSH, SOD, and Trx levels (Fig. 5c). Fig. 5d showed the western blotting result. These results confirmed that Vit C exerted protective effect against Cr (VI)-induced hepatotoxicity in the L-02 hepatocytes.

### Vit C protected against Cr (VI)-induced hepatotoxicity in vivo

**Fig. 6:**
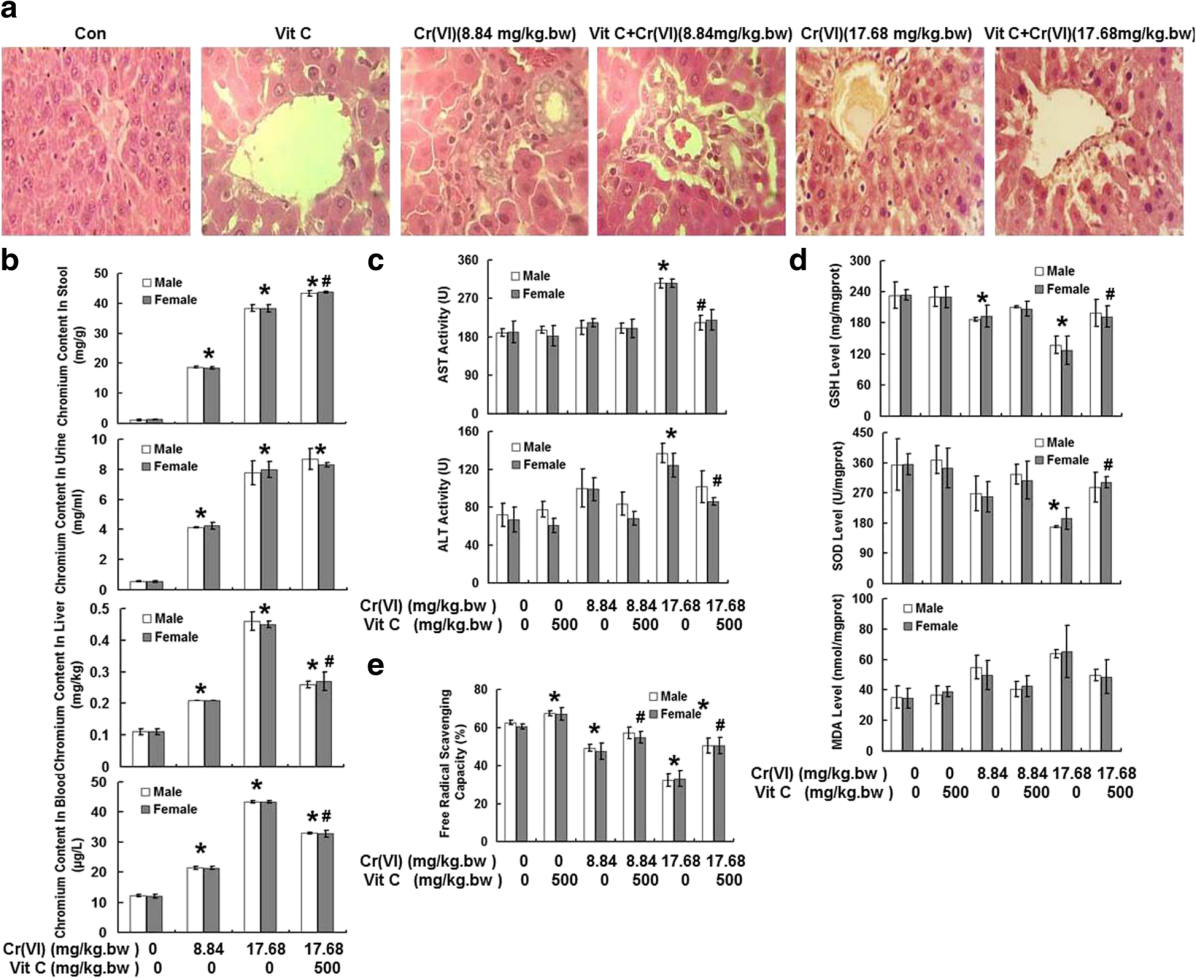
Vit C protected against Cr (VI)-induced hepatotoxicity in vivo. All rats were given the drugs by gavage at a dose of 0.5 ml/100 g body weight daily for a week (seven consecutive days). Groups are indicated by pretreatment + treatment as follows: Con, Vit C, Cr (VI) (8.84 mg/kg.bw), Vit C+ Cr (VI) (8.84 mg/kg.bw), Cr (VI) (17.68 mg/kg.bw), and Vit C+ Cr (VI) (17.68 mg/kg.bw). **a** Effect of Vit C pretreatment on Cr (VI)-induced alterations in rat liver histology. **b** The chromium contents in stool, urine, liver and plasma. **c** AST and ALT activities. **d** GSH, SOD, and MDA levels. **e** Free radical scavenging capacity. Data represent mean ± SD. *p < 0.05, compared with control group. ^#^ *p* < 0.05, compared with the Cr (VI) alone treatment group

All rats were given the drugs by gavage at a dose of 0.5 ml/100 g body weight daily for a week (seven consecutive days). Groups are indicated by pretreatment + treatment as follows: Con, Vit C, Cr (VI) (8.84 mg/kg.bw), Vit C+ Cr (VI) (8.84 mg/kg.bw), Cr (VI) (17.68 mg/kg.bw), and Vit C+ Cr (VI) (17.68 mg/kg.bw). As shown in Fig. 6a, a microscopic examination of the liver samples from all treatment groups revealed the typical histopathological features. Both the control and the Vit C (500 mg/kg.bw) group represented the normal rat liver showing normal hepatic architecture (H & E, ×200 magnification). The administration of Cr (VI) (8.84 mg/kg.bw) induced focal necrosis in the centrilobular region with infiltration of neutrophils and lymphocytes, while the hepatic lobules were clear and the hepatic cords were arranged in order. The histology of the livers from Vit C (500 mg/kg.bw) pretreatment plus Cr (VI) (8.84 mg/kg.bw) group showed slight inflammatory cell infiltration. And the Cr (VI) (17.68 mg/kg.bw) group revealed moderate to intense cytoplasmic vacuolization, central vein stenosis, and hepatocyte focal necrosis. Vit C (500 mg/kg.bw) pretreatment significantly alleviated Cr (VI) (17.68 mg/kg.bw)-induced pathological changes. We also examined the effect of Vit C on Cr excretion and Cr content in plasms and liver. Fig. 6b revealed that treatment of Cr (VI) significantly increased Cr content in stool, urine, liver and plasma in a dose-dependent manner. Vit C pretreatment plus Cr (VI) (17.68 mg/kg.bw) group showed higher fecal excretion and lower Cr content in liver and plasma compared with that of the Cr (VI) (17.68 mg/kg.bw) alone treatment group, indicating that Vit C treatment accelerated the fecal excretion of Cr in liver and plasma. High dose of Cr (VI) treatment increased AST activity, and Vit C pretreatment only alleviated high dose of Cr (VI)-induced AST activity elevation. Both low and high dose of Cr (VI) treatments increased ALT activity, and Vit C pretreatment showed obvious inhibitory effect on AST activity elevation (Fig. 6c). The examination of effect of Vit C on Cr (VI)-induced hepatic antioxidant system damage showed that Cr (VI) decreased GSH and SOD levels and increased MDA level, and Vit C pretreatment reduced the antioxidant system damage (Fig. 6d). MDA quantification is known as the most widely used method to evaluate lipid peroxidation, the general mechanism accounts for cell injury and other cellular toxic ending. Cr (VI) inhibited free radical scavenging capacity, and Vit C pretreatment also showed the protective effect, suggesting that Vit C rescued the decrease of Cr (VI)-induced free radical scavenging capacity (Fig. 6e). Male and female rats showed similar results. Our present data revealed Vit C pretreatment effectively inhibited Cr (VI)-induced hepatotoxicity in the rat model, and the protective effect of Vit Cwas associated with the inhibition of various enzymatic markers and cytokines as well as the restoration of the antioxidant system function.

## Discussion

It is known that heavy exposure to Cr (VI) is closely associated with the increased risk of liver primary cancers [13], thus study on the hepatotoxicity induced by Cr (VI) has become the hot spot in the research field of toxicology. Liver is particularly susceptible to injury for its involvement in xenobiotic metabolism. Under resting conditions the hepatocytes must maintain the critical balance between cellular oxidants and antioxidant defenses. The disruption of this balance may cause the entrance of the cells to an inflammatory state, resulting in the damage of both the cells involved and the surrounding tissues due to the induction of the inflammatory cytokines, the activation of various signaling pathways, and other molecular and cellular modifications. Inflammation is known as the protective mechanism to help the injured or infected organism to initiate cellular repair processes and to restore physiological functions. Liver inflammation has been shown to be associated with elevated production of various cytokines such as IL-1β, TNF-α and IFN-γ, which have been implicated in hepatocarcinogenesis [14, 15]. Although the activities of AST and ALT are the most commonly used and well-known enzymatic markers of liver injury, they only change in late stages and often lack sensitivity in early stages of various liver diseases [16]. IL-1β is known as an initiator cytokine that plays an important role in the regulation of the inflammatory responses [17]. As a major endogenous mediator of hepatotoxicity, TNF-α is a pro-inflammation cytokine that expresses in various liver injuries and plays an important role in tissue damage. Being another pro-inflammatory cytokine, IFN-γ is also a sensitive biomarker as well as a critical mediator of liver damage from several xenobiotic agents [18]. IL-10 is known to have anti-inflammatory effect for its ability to down-regulate the production of pro-inflammation cytokines such as IL-1β and IFN-γ from T cells and exerts its inhibitory effect on several model of liver injury [19]. We found IL-10 level was increased at low dose of Cr (VI) treatment but decreased at high dose of Cr (VI) treatment, indicating that IL-10 played a compensatory role at first, but then showed compensatory failure in severe liver injury. The data indicates that liver damage is likely occurring at the high dose of Cr (VI) treatment because we can speculate that the liver may be able to recover from the injury and regain its normal functions at low dose of Cr (VI) treatment. LTB4, which can be synthesized on activation of 5-lipoxygenase (5-LO), has also been confirmed to participate in different experimental models of liver injury [20].

Cr (VI) could enter the target cells and then undergoes metabolic reduction to Cr (III), causing the accumulation of ROS together with a cascade of various cellular toxic events. MRCC I is a large enzyme complex that embedded in the inner mitochondrial membrane and plays an important role in energy metabolism by proving proton-motive force required for ATP synthesis [21]. While other studies have confirmed that MRCC III is the important source of cellular ROS, in the present study we showed that MRCC I may be the precise site for ROS generation after Cr (VI) exposure. Complex I consists of at least 45 subunits of which 38 subunits are encoded by nuclear genome and 7 are encoded by the mitochondrial genome [22]. In order to investigate how Cr (VI) inhibits MRCC I, we checked the expression levels of all the subunits involved in MRCC I assembly by performing gene chip and RT-PCR. Data revealed that Cr (VI) significantly affected NDUFS3. The mechanisms involved in Cr (VI)-induced inhibition of MRCC I remain to be fully explored. There is evidence supporting that in hepatocytes, the pro-inflammatory cytokines such as TNF-α and IFN-γ can also induce ROS accumulation [23], but the related mechanism is not clear. ROS exhibit the dual role in biology. When produced by normal cellular metabolism and in limited quantities, ROS exert beneficial effects on mediating signaling pathways and contributing to cellular functions including proliferation and differentiation. However, the over-generation of ROS may act as key players in disease pathogenesis and induce cell and tissue damage by attacking vital cellular components such as DNA, lipids and proteins. OS is the state which can result from the increased formation of ROS and the unbalance between pro-oxidants and antioxidants [24]. Chaverrí et al. has reported that OS is associated with Cr (VI)-induced nephrotoxicity [25]. ROS scavenging enzymes, including SOD and GSH, are the members of antioxidants system whose function is to eliminate excess ROS. SOD is known to facilitate the conversion of superoxide to hydrogen peroxide. And GSH is a tripeptide responsible for protection against free radicals, and the depletion of GSH could decrease cellular antioxidant capacity and induce oxidative stress. It has been reported that infection with hepatitis C is accompanied with the accumulation of ROS and the inhibited antioxidant levels [26], thus we inferred that the decreased levels of antioxidant defenses, which were characterized by the inhibition of GSH, SOD and Trx levels, together with the augmented formation of ROS, appear to play an important part in Cr (VI)-induced liver injury. The role of free radicals in Cr (VI)-induced hepatotoxicity and the capacity of Cr (VI) to promote OS are important areas of research in toxicology, because such information may possess important therapeutic significance to prevent liver injury even cancer progression after Cr (VI) by antioxidants such as Vit C.

Previous reports suggest that Cr (VI) is a hepatotoxin and Cr (VI)-induced hepatotoxicity can be alleviated by several natural and synthetic compounds [27]. We think that free radical accumulation and the occurrence of OS is early event and the main mechanism of Cr (VI)-induced liver damage, thus the administration of antioxidant, especially in the early stage of Cr (VI) exposure, may significantly diminish liver injury and even inhibit hepatocarcinogenesis. The present research we conducted in vivo study. The purpose of utilization of experimental rat model of toxicant-induced hepatotoxicity is to evaluate the biochemical processes involved in various liver diseases and to explore the possible pharmacological effects of the liver protective agents such as Vit C. Based on our results, pretreatment with Vit C inhibited the above-mentioned hepatotoxicity-related alterations both in vitro and in vivo, and accelerated the fecal excretion of chromium in liver and plasma of the rates, indicating the hepatoprotective effect of Vit C against Cr (VI)-induced liver injury. Considering the difference between animals and humans and before we can provide valuable experimental evidence for the anti-oxidative therapy in clinic, we definitely need conduct further study because the effective dose and safe dose, during of treatment, and bio-availability of Vit C require thorough exploration.

The current federal maximum contaminant level for total Cr is 100 μg/l [28]. A 2-year cancer bioassay conducted by the National Toxicology Program (NTP) reported that administration of Cr (VI) in drinking water (in the form of sodium dichromate dihydrate [SDD]) induced tumors in the small intestines of rats at ≥172 mg/l SDD (≥60 mg/l Cr (VI)) [29]. The increasing evidence has suggested that both inflammation and ROS play important role in the induction of the carcinogenic phenotype. It is confirmed that inflammatory cell infiltration during cancer progression is accompanied with the generation of various cytokines, chemokines and growth factors, favoring increased cellular proliferation [30]. ROS generated from ETC and oxidation-reduction system after Cr (VI) exposure could cause oxidative damage to host DNA, resulting in activation of oncogenes and/or inactivation of tumor suppressor genes as well as various epigenetic modifications that favor tumor progression. Although we focused on liver injury and the protective effect of Vit C after Cr (VI) exposure in vitro and in vivo, the present study also provided important experimental evidence for the mechanism and treatment study of Cr (VI)-associated cancers. And in addition to the exploration of Cr (VI)-induced cytotoxicity and carcinogenicity, future attention should also be paid to the development of antioxidant-based strategies for primary prevention of liver injury even primary liver cancers in occupational Cr (VI) exposure individuals.

## Conclusions

The present study confirmed that ROS played a role in Cr (VI)-induced hepatotoxicity and Vit C exhibited protective effect. Our current data provides important clues for studying the mechanisms involved in Cr (VI)-induced liver injury, and may be of great help to develop therapeutic strategies for prevention and treatment of liver diseases involving ROS accumulation for occupational exposure population.

## Abbreviations

5-LO: 5-lipoxygenase
ALT: alanine aminotransferase
AST: aspartate aminotransferase
Cr (VI): hexavalent chromium
Cyt c: cytochrome c
ETC: electron transfer chain
GSH: glutathione
IFN-γ: interferon-γ
IL-10: interleukine-10
IL-1β: interleukine-1β
LTB4: leukotriene B4
MDA: malondialdehyde
MRCCs: mitochondrial respiratory chain complexes
NADH: Nicotinamide adenine dinucleotide
NADPH oxidase: nicotinamide adenine dinucleotide phosphate-oxidase
ROS: reactive oxygen species
SOD: superoxide dismutase
TNF-α: tumor necrosis factor-α
Trx: thioredoxin
Vit C: Vitamin C
